# Experimental evidence for the preservation of U-Pb isotope ratios in mantle-recycled crustal zircon grains

**DOI:** 10.1038/s41598-018-30934-4

**Published:** 2018-08-27

**Authors:** Fernando Bea, Pilar Montero, Jose Francisco Molina Palma

**Affiliations:** Department of Mineralogy and Petrology, Campus Fuentenueva, University of Granada, 18002 Granada, Spain

## Abstract

Zircon of crustal origin found in mantle-derived rocks is of great interest because of the information it may provide about crust recycling and mantle dynamics. Consideration of this requires understanding of how mantle temperatures, notably higher than zircon crystallization temperatures, affected the recycled zircon grains, particularly their isotopic clocks. Since Pb^2+^ diffuses faster than U^4+^ and Th^+4^, it is generally believed that recycled zircon grains lose all radiogenic Pb after a few million years, thus limiting the time range over which they can be detected. Nonetheless, this might not be the case for zircon included in mantle minerals with low Pb^2+^ diffusivity and partitioning such as olivine and orthopyroxene because these may act as zircon sealants. Annealing experiments with natural zircon embedded in cristobalite (an effective zircon sealant) show that zircon grains do not lose Pb to their surroundings, although they may lose some Pb to molten inclusions. Diffusion tends to homogenize the Pb concentration in each grain changing the U-Pb and Th-Pb isotope ratios proportionally to the initial ^206^Pb, ^207^Pb and ^208^Pb concentration gradients (no gradient-no change) but in most cases the original age is still recognizable. It seems, therefore, that recycled crustal zircon grains can be detected, and even accurately dated, no matter how long they have dwelled in the mantle.

## Introduction

The discovery of older-than-host zircons of crustal origin in modern Mid-Atlantic Ridge oceanic gabbros by Pilot *et al*.^1^ caused a significant impact in the Earth Science community - it also caused skepticism. Not all scientists accepted that the zircons were crustal and suggested that they might have resulted from contamination either during rock processing for mineral separation or with drilling muds, a possibility recently demonstrated by Andrews *et al*.^2^. At around the same time as the discovery of the ocean gabbros zircons, geologists working in Kytlym, a concentrically zoned ultramafic body of the Urals Platinum-Bearing Belt^3^, also found crustal zircon in the dunites forming the core of the massif. The discovery was again attributed to contamination despite tiny grains of a diamond-like mineral being observed in some hand specimens. To clarify the conundrum, we processed two kilograms of carefully cleaned dunite boulders at the University of Granada. The boulders were crushed between two steel plates with a hydraulic press and then manually in a steel mortar. To avoid any contamination from previously processed samples all the crushing material used was new. The resulting fragments (Ømax < 1 mm) were dissolved by repeated application of HF over two months following the method described by Neuerburg^4^. This produced a residuum with several zircon grains, most of them older than the 330 Ma dunite (410 Ma to 2800 Ma), with elevated U and Th and cathodoluminescence images typical of granitic zircons, that we concluded were xenocrysts of crustal origin^5^. Ongoing oxygen isotope work fully confirms that conclusion.

Following these initial findings, crustal zircons often accompanied by other crustal minerals have been found in a wide variety of mantle-derived rocks. The catalog includes Mid-Atlantic basalts and gabbros^6,7^, ophiolites^8–11^, and supra-subduction magmas^12–16^. It may also include orogenic lherzolites such as Ronda^17^ and Finero^18^.

These findings pose two questions with broad geodynamic implications: (i) which path have these zircons followed from the continental crust to the mantle? (ii) how have they survived transport through the mantle with their isotopic systems apparently undisturbed? To date, neither of these question has been satisfactorily resolved. Recognition of zircon xenocrysts in mantle-derived rocks is mainly based on the discrepancy between the zircon U-Pb and the whole-rock age. So it is of primordial importance to understand how thermal shock during transport into the mantle affects the zircon U-Th-Pb isotope ratios. In nature U and Th ions are always tetravalent, but Pb has two oxidation states, 4+ and 2+, the latter forming the most stable compound because of the “inert-pair effect” that affects the heaviest elements of the B subgroups. The diffusivity of Pb^4+^ is similar to U^4+^ and Th^4+^ but Pb^2+^ diffuses much faster. Accordingly, it is essential to determine the Pb oxidation state in zircon. Watson *et al*.^19^ proposed that it is Pb^2+^ but Kramers *et al*.^20^, to explain the scarcity of observable strong diffusion effects in pre-magmatic zircons (see discussion in^21^), suggested that it is tetravalent. The latter, however, found no support from XANES measurements because these revealed that Pb^2+^ is far more abundant than Pb^4+^ ^22^. It seems, therefore, that either radiogenic Pb is mostly generated as Pb^2+^ or, if some is generated as Pb^4+^, it is reduced to a divalent state due to the strong reducing environment of the zircon^23^.

Accepting this, Arrhenius equations^24–26^ indicate that reheating at T > = 1000 °C increases the Pb^2+^ diffusion coefficient to values at which it may easily migrate outwards from the zircon crystal (D ≈ 3.37 * 10^−24^ m^2^s^−1^ at 1000 °C; D ≈ 6.2 * 10^−20^ m^2^s^−1^ at 1300 °C). The U^4+^ and Th^4+^ diffusion coefficients are about 5 to 7 orders of magnitude lower, thus implying that diffusion can substantially disturb the U-Th-Pb isotope ratios of zircon xenocrysts, especially those recycled through the mantle because of the long timescales and high temperatures involved.

**Figure 1:**
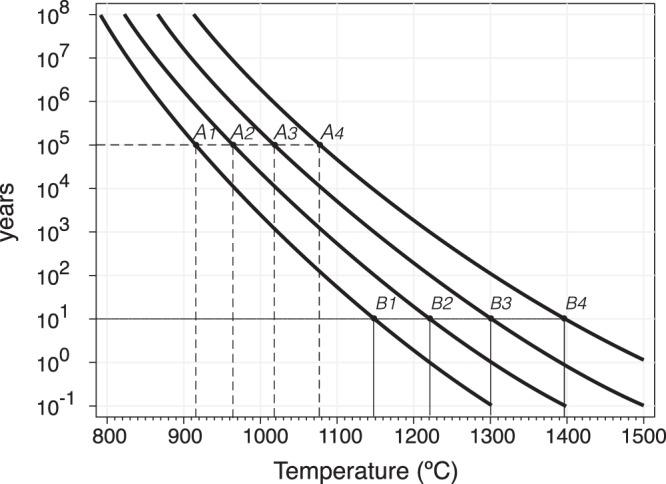
Time-temperature equivalences for Pb diffusion in zircon. The t-T coordinates along the same line result in identical diffusion profiles across two domains with different Pb concentration. The only difference between the four curves is they are suitable for different t-T combinations. For example, heating at 915 °C for 10^5^ years (point A_1_) is equivalent to heating at 1138 °C for 10 years (point B_1_), or heating at 1177 °C for 10^5^ years (point A_4_) is equivalent to heating at 1396 °C for 10 years (point B_4_). Calculations were done with the COMSOL^TM^ ^58^ software using Cherniak and Watson Arrhenius equation: DPb = 1.1 × 10^−1^ exp^(−550±30 kJ mol−1/RT)^ m^2^sec^−1^ ^1,2^.

The thermally enhanced Pb^2+^ diffusion may produce two extreme effects depending on the diffusive behavior of the zircon environment: (i) progressive loss of radiogenic Pb from the zircon to the outside ultimately resetting the U-Th-Pb geochronometers (ii) homogenization of the Pb concentration throughout the crystal with no Pb lost from the zircon grain. Most researchers only consider the first effect. Pilot *et al*.^1^, for example, proposed that under shallow mantle conditions (~1000 °C) zircon grains would totally lose their radiogenic Pb in less than 150 million years. This would set an upper limit for mantle residence time during which older-than-host zircon could be detected. Moreover, considering that temperature increases with depth and diffusivity increases exponentially with the temperature (Fig. 1) prevalence of the Pb loss effect implies that crustal zircon grains would not be detected if they were carried to greater depths with temperatures ≥1200 °C (90 to 130 km depending on the geotherm). In these conditions Pb would be lost in less than 0.05 Myr from crystals with sizes similar to the grains found in common rocks.

Yet, the above estimations might be incorrect because they implicitly assume that the material hosting zircon has much higher Pb^2+^ diffusivity and partitioning than the zircon itself, and consequently may behave as a Pb^2+^ sink. Certainly, a zircon grain surrounded by a high-diffusivity material, such as silicate melt, will be quickly depleted in Pb (or, more probably, dissolved before diffusion may cause perceptible effects, see Figure 14 in^21^). However, if zircon xenocrysts are surrounded by, or included in, minerals that hardly accept Pb, i.e., with very low Pb partitioning such as olivine^27,28^, orthopyroxene^29^, etc., the Pb loss from zircon grains might be insignificant and age resetting never occurs.

The objective of this paper is to understand whether crustal zircon xenocrysts extracted from mantle-derived rocks can yield reliable U-Th-Pb ages, which is the essential premise to track their origin and understand how they were recycled in the mantle. To this end, we studied the diffusive behavior of the U-Th-Pb system in natural unaltered zircon grains extracted from two rocks: a Variscan tonalite (SAB51) with chemically zoned but isotopically homogeneous zircon precisely dated at 318 ± 2 Ma; and a Cambrian-Ordovician orthogneiss (SAB50) in which most zircon grains have a ca. 480 Ma–485 Ma rim and a ca. 600 Ma- 610 Ma core^17^. The former was selected to consider how Pb diffusion affects isotopically homogeneous but chemically heterogenous zircon grains. The latter was selected to consider how diffusion affects zircon grains with different age components.

## Experimental Procedure

To determine the effects of diffusion we heated several sets of zircon grains from the two samples mentioned before at different temperature and over different times. These heated grains were then analysed for U-Th-Pb isotopes with the IBERSIMS SHRIMP ion microprobe. The results were compared to unheated pristine zircon grains from the same rocks and it was assumed that the differences resulted solely from diffusion during the experiments.

Given the long time involved in geological processes and the necessarily short duration of experiments, diffusion in laboratory conditions needs the heating to be at much higher temperatures to produce comparable effects (Fig. 1). Unfortunately, the experimental temperature is limited by the thermal decomposition of zircon, which may hinder, or even prevent, work with natural specimens. At atmospheric pressure pure ZrSiO_4_ decomposes to SiO_2_ + ZrO_2_ at 1673 ± 10 °C^30^, but the transformation occurs at much lower temperature, around 1400 °C, if: zircon contains impurities^31^; or reacts with a silica-subsaturated phase such as forsteritic olivine to form baddeleyite + orthopyroxene^32^. Váczi *et al*.^33^ determined that natural zircon grains heated in open crucibles at 1400 °C for just 96 hours always partially decompose, and that the crucible material exerts a great influence on the zircon behavior. We have found that, after one week, fresh natural zircon grains embedded in forsterite begin decomposing at around 1300 °C, and at 1400 °C if embedded in orthopyroxene or garnet. They, however, may remain stable for months at temperatures up to 1500 °C if embedded in microcrystalline silica (cristobalite at experiment temperatures), probably because the excess SiO_2_ suppresses the reaction zircon = baddeleyite + silica. This, therefore, permits higher experimental temperatures to be reached than when the zircon is embedded in olivine, pyroxene or garnet. The microcrystalline silica additionally provides an environment of very low Pb^2+^ partitioning, analogous in this regard —and only in this regard— to mantle olivine or orthopyroxene.

Accordingly, we loaded zircon concentrates of about 100 grains from each rock type embedded in pure microcrystalline silica and heated them in small alumina crucibles in a N_2_ atmosphere at: 1300 °C for 30, 90, and 180 days; and at 1500 °C for 30 days.The last conditions being roughly equivalent to 1 yr at 1300 °C (Fig. 1, see Supplementary Material). The microcrystalline silica was prepared by heating smoked silica in a platinum crucible at 1400 °C for 24 h and then grinding the product, mostly cristobalite, in an agate mortar to a grain size less than 10 µm. This ensured maximum contact between zircon grains and the silica host. After each heating experiment, zircon grains were recovered by dissolving the silica in warm HF, first washing it with a saturated H_3_BO_3_ solution and then with ultrapure water to eliminate all traces of the acid. The so released zircons grains were dried, mounted in epoxy, documented with the SEM, and analyzed with the SHRIMP as described in the Samples and Methods section. To check whether some Pb had migrated from zircon to the enclosing cristobalite, one run (1400 °C/ 90 days) was vacuum impregnated in epoxy, cut in several slices, and the silica grains surrounding the zircon analysed with a QP-LACIPMS system. In all cases Pb was below detection limit thus indicating that cristobalite acted as an effective Pb sealant.

## Results

**Figure 2:**
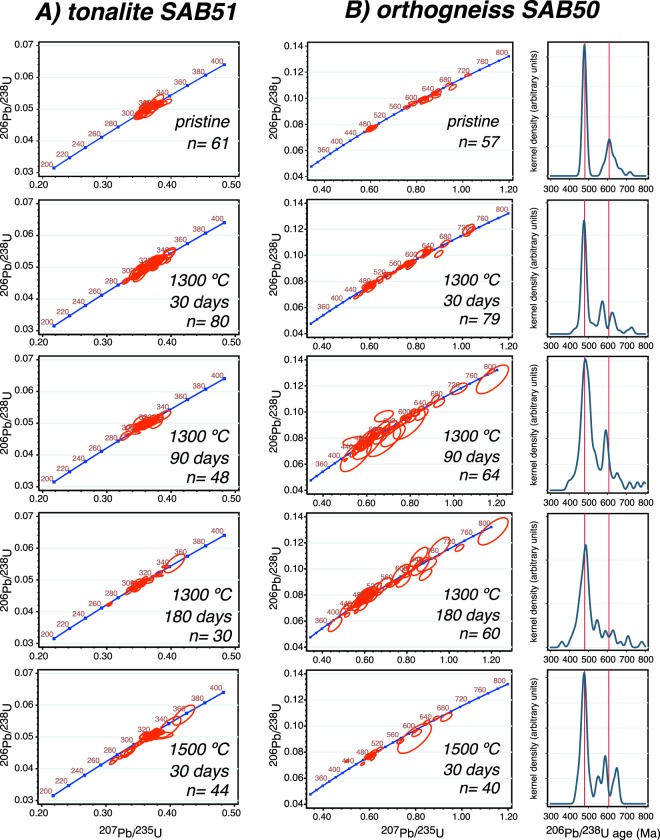
Experimental results. (**A**) Wetheril concordia plots of the heated zircon grains from the tonalite SAB51. (**B**) Wetheril concordia plots and distribution of ^206^Pb/^238^U ages of the heated zircon grains from the orthogneiss SAB50. Note how the spots representing the tonalite SAB51 spread up and down with increasing heating time and/or temperature producing both, direct and reverse discordant ages. The orthogneiss SAB50 shows the same effect in the rims, and a loss of definition in the age of the cores, with the appearance of new modes corresponding to mixed ages.

**Figure 3:**
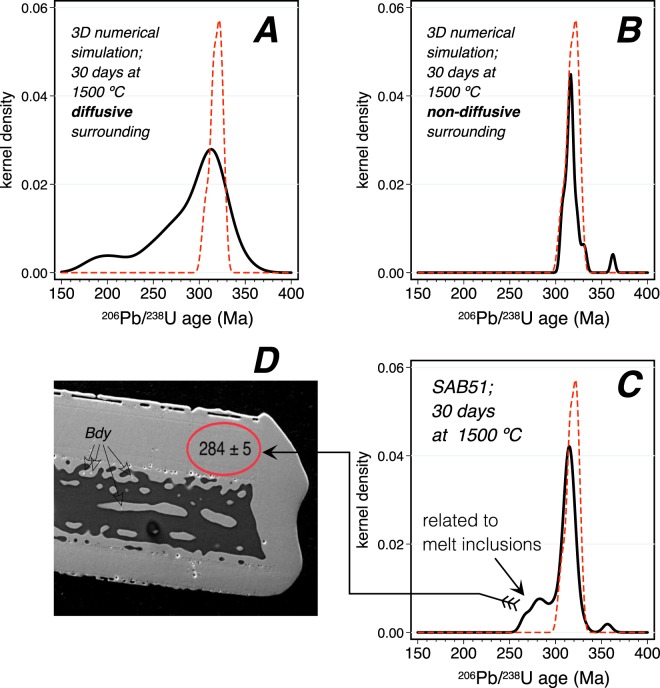
(**A**) Numerical simulation of diffusion in SAB51 at 1500 °C over 30 days in a diffusive surrounding. (**B**) idem in a non-diffusive surrounding. (**C**) Age distribution in the experimental run at 1500 °C for 30 days. The red dashed line represents the distribution of the pristine SAB51 zircon grains. Note the excellent match between the experiment and the simulation with non-diffusive surrounding, except for some data that yielded ^206^U/^238^Pb ages between 250 Ma to 300 Ma. These were always found around melt inclusions. (**D**) Secondary electron image of a zircon grain with a large molten albite inclusion. New zircon and some baddeleyite (Bdy) are produced at the zircon melt interface. The red ellipse represents the spot analyzed with the SHRIMP, which showed loss of lead to the inclusion (also see Supplementary Material).

Figure 2A shows the Wetherill concordia plots of pristine and heated zircon grains from the Variscan tonalite SAB51 (Table 1 in Supplementary Material). Pristine zircon grains define a neat cluster of concordant ages with a mean of 318.6 ± 1.5 Ma. Zircon grains heated at 1300 °C for 30 days perceptibly spread up and down along concordia, this effect increases with time and temperature to reach a maximum in the 1500 °C/30 days experiment. Figure 3 compares these results to two alternative 3D numerical simulations of Pb diffusion for the same T/t conditions, one considers a diffusive surrounding, similar to a melt, and the other a non-diffusive surrounding about three orders of magnitude less diffusive than zircon. The experimental results do not fit well with the simulation that considers a diffusive surrounding, because this will cause tremendous Pb loss resulting in highly discordant and very low U-Pb ages (Fig. 3A). The experimental results do, however, fit well with the simulation that considers a non-diffusive environment (Fig. 3B) except for a small fraction of the experimental data with younger ^206^U/^238^Pb ages between 250 Ma to 300 Ma peaking at about 285 Ma (Fig. 3C). Remarkably, these slightly discordant ages always correspond to spots analyzed in the region of large inclusions of albitic glass (Fig. 3D) presumably representing molten inclusions of albite (melting point ≈1100 °C at 1 atm^34^), that are very abundant in the pristine SAB51 zircon grains. The inclusions apparently act as local Pb sinks enhancing diffusion and removing Pb from the zircon so lowering the apparent U-Pb age.

**Figure 4:**
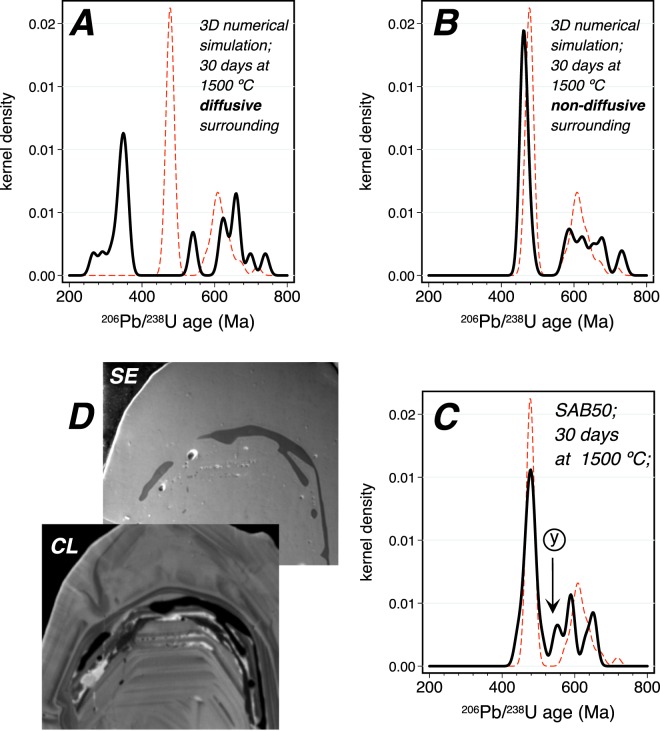
(**A** and **B**) Numerical simulation of diffusion in SAB50 at 1500 °C over 30 days in diffusive and non-diffusive surroundings. Note the huge displacement of the rims peak in the former. (**C**) Age distribution of the experimental run at 1500 °C for 30 days. The red dashed line represents the distribution of the pristine SAB50 zircon grains. Again, the experiment and the simulation with non-diffusive surrounding match well, except for the appearance of the peak “Y”, which correspond to analyses in the region of melt inclusions. (**D**) Secondary electron (SE) and cathodoluminescence (CL) images of a zircon grain with an accumulation of albitic glass at the interface between the Cambro-Ordovician rim and the Ediacaran core (see text).

Figure 2B shows the Wetherill concordia and age distribution plots for the orthogneiss SAB50 zircon grains (Table 2 in Supplementary Material). Pristine unheated zircon grains show two well-defined modes: a narrow peak at ca. 480 Ma that corresponds to the rims, and a wider peak at ca. 605 Ma that corresponds to the cores. A few grains contain older cores not shown in the figure. On heating, the 480 Ma mode widens but the peak position remains constant. The 605 Ma mode, in contrast, is replaced by several randomly distributed small peaks so that the 605 Ma age becomes unrecognizable. Comparing the 1500 °C/30 days experiment to 3D numerical simulations shows again that the experimental data do not fit with the simulation that considers a diffusive surrounding because this produces highly discordant rim ages peaking at ca. 380 Ma, far from the true mode (Fig. 4C). On the other hand, the simulation with a non-diffusive surrounding closely matches the experiments, except for the peak labeled “Y” in Fig. 4B, which can also be attributed to some Pb loss by diffusion into small bands of, mostly albitic, glass. These glass bands generally appear at the rim-core interfaces (Fig. 4A) filling microcracks that were most likely caused by the non-uniform thermal expansion of the rim-core systems (see^35^ and references therein).

## Discussion and Conclusions

Extrapolation of the experimental results to mantle recycling of crustal zircon grains requires understanding first whether or not these dwelled in the mantle surrounded by a diffusive or non-diffusive environment and, second, whether the experimental conditions, especially silica embedding, adequately simulate the environment for zircon grains included in mantle minerals.

Regarding the second question, we must consider the following: The few available data about Pb in natural quartz indicate concentrations at the sub-ppb level (e.g.^36^). Our own LA-ICPMS measurements, even in quartz crystals coexisting with Pb-rich feldspars are always below detection limit (≈1 ppb). This indicates that the incorporation of Pb^2+^ impurities into quartz crystals is very limited, as expected from the inverse relation between impurities uptake and ionic radius show by Chakraborty^37^. Unfortunately, the Pb^2+^ diffusivity in silica polymorphs has not been studied. From the varied experimental data summarized in^38^ it follows that diffusivity decreases with increasing ionic radius for alkaline elements, for example Ca^2+^ (Ø ≈ 1.12 Å) diffusivity is orders of magnitude slower than Na^+^ (Ø ≈ 1.18 Å). It would be logical to expect, therefore, that Pb^2+^ (Ø ≈ 1.29 Å) would diffuse much slower than Ca^2+^. The combination of very low Pb uptake and low diffusivity causes silica embedding to be an effective Pb sealant. This is supported by the above mentioned fact that the Pb concentration in silica grains surrounding zircon after a 1400 °C/90 days experiments always had Pb below LA-ICPMS detection limit.

Regarding the first question, it should be noted that mafic and ultramafic melts have elevated Pb^2+^ diffusivities^39^ but they are extremely subsaturated in zircon^40,41^.They are capable, therefore, of dissolving all zircon grains in contact with them almost instantaneously, in fact much before diffusion might cause perceptible effects (see Fig. 14 in^21^). We must assume, therefore, that crustal zircon recycled in the mantle could only survive if they are shielded from the melt, i.e., included in mantle minerals. Pb^2+^ is highly incompatible in olivine, orthopyroxene and garnet, moderately incompatible in spinel and clinopyroxene, and compatible in plagioclase and apatite^42–44^. Compared to zircon, Pb^2+^ diffusion is faster in clinopyroxene, plagioclase and apatite^45–47^, of the same order in orthopyroxene^47^, no data are available for olivine and aluminosilicate garnets. Nevertheless, the very low Pb^2+^ partitioning into olivine, orthopyroxene, and garnet suggests that these minerals will likely seal zircon xenocrysts included in them so preventing the migration of Pb out of the zircon grain. In contrast, plagioclase, apatite and, to some extent, spinel and clinopyroxene will not have the same effect.

Plagioclase is limited to the uppermost mantle layer; spinel and apatite are scarce, so these three minerals are unlikely to host many recycled zircon grains. Therefore, with the probable exception of zircon grains included in clinopyroxene, most zircon xenocrysts would be enclosed by minerals that act as a barrier for the diffusive Pb migration loss, i.e., similar in this regard to our experimental conditions.

If this is the case, our results indicate that most zircon grains do not lose radiogenic Pb and thus never fully reset their isotopic clocks. Lead diffusion may occur within each zircon crystal and tends to homogenize the Pb concentration. Even for highly heterogeneous zircon grains such as from the orthogneiss SAB50 homogenisation occurs very quickly, in about 500 years at 1300 °C, and 3 * 10^5^ years at 1100 °C (Fig. 1). This effect modifies the U-Pb and Th-Pb isotope ratios to a level that solely depends on the initial U, Th and radiogenic Pb distributions^21^. This situation, however, can be slightly modified if recycled zircon grains contain large inclusions of glass, albite or other easy-to-melt material. If the temperature is high enough for these to fuse, the so-formed liquid may form streaks or pockets that act as finite Pb sinks, thus depleting the surrounding zircon and causing localized slightly discordant U-Pb ages.

It might be argued that our experimental results do not account for the structural state of recycled zircon grains, which may range from crystalline to metamictic. However, as summarized in^48^, moderately radiation-damaged zircon grains completely reorganize their structure at 900 °C in matter of hours or days, becoming highly crystalline before diffusion may cause any effect. This may causes atom-scale reorganization of Pb^49^ and even the formation of metallic Pb nanospheres^50^. These effects may certainly degrade the quality of SIMS measurements, but the effects do not seem to be significant. Heavily damaged or amorphous zircon grains, on the other hand, will segregate into their constituent oxides^48^ [and references therein] so that they are not found as inherited zircons.

In summary, recycled crustal zircon grains that were initially homogeneous or nearly homogeneous will yield accurate U-Pb crystallization ages, independent of how long they have remained in the mantle. Initially heterogeneous zircon grains, on the other hand, would show a spread of subconcordant ages along concordia, reverse discordias, and spurious high U-Pb ages as shown in the numerical models of^21^.

Our results also indicate that Pb loss because of diffusion imposes no limit on the depth that recycled zircon grains may reach; depth is only limited by the zircon transformation to the high-density polymorph reidite. According to the experimental data of Ono *et al*.^51^, this occurs at P (GPa) = 8.5 + 0.0017 × (T − 1200) in the temperature range 1100–1900 K and sets the upper stability limit of zircon in the mantle at ~250–270 km if zircon crystals are shielded from any melt present.

## Samples and Methods

### Samples

For this work we selected two samples containing abundant unaltered zircon with no, or very little, common lead. The first sample, SAB51, is a Variscan tonalite from the Sanabria zone, in NW Iberia, which contains abundant zircon grains. Most of them are long narrow prisms (150–300 µm) terminated by short pyramids, transparent, colorless to pinkish, and contain abundant inclusions, mostly albite. Previous SHRIMP work (authors’ unpublished data) revealed that SAB51 zircon grains are isotopically homogeneous, with a U-Pb age of 318 ± 2 Ma, but chemically heterogeneous, with U ranging from about 230 ppm to 1700 ppm.

The second sample, SAB50, is a Cambro-Ordovician orthogneiss from the Ollo de Sapo formation of the Central Iberian Zone^52^. Rocks from this formation have an abnormally high zircon inheritance so that about 90% of their zircon grains consist of ca. 485 Ma rims around ca. 605 Ma, in some cases older, cores^52–55^. The sample studied here is medium-grained, with a marked augen structure defined by large K-feldspar crystals, and is slightly metamorphosed under lower greenschists facies. It contains abundant zircon grains. Most of the crystals are short stubby prisms terminated at both ends by long pyramids with total length between 100 and 250 µm. The crystals are transparent, pinkish, with small inclusions and optically noticeable ellipsoidal cores occupying most of the central prismatic body.

In both cases, zircon concentrates were separated from about 15 kg of crushed fresh rock sieved between 300 µm and 50 µm. Separation was done by panning, first in water and then in ethanol. The concentrates were purified first with an Nd magnet, and then by hand picking to remove all traces of other minerals.

Batches of 60–80 grains of each rock were repeatedly analyzed with the SHRIMP before starting the diffusion experiments to ensure they always yielded the same age distribution, i.e., the initial heterogeneity of the zircon loads does not mask the effect of heating. Different batches of the tonalite SAB51 zircon grains always yielded the same age. Different batches of the orthogneiss SAB50 always yielded the same age in the rims but some minor differences in the cores caused by variable, but always small, fraction of older-than-Ediacaran cores. This variation is not relevant for the experiments because only those grains with cores younger than 800 Ma were considered.

### Diffusion experiments

Volume diffusion in zircon is practically insensitive to pressure, for this reason the experiments were carried out in open crucibles within a N_2_ atmosphere using the following procedure: cylindric alumina crucibles with a diameter of 12 mm and height of 14 mm were filled with a 5 mm thick bed of fine-powered microcrystalline silica. This was mainly cristobalite, obtained by heating smoked silica at 1500 °C for 3 days in a platinum container and grinding the resulting product in an agate mortar. About 100 zircon grains were then transferred on top of the silica layer using a needle. The zircon grains were covered with another 5 mm thick silica layer, and the whole load was then pressed with a steel piston. The crucibles were placed in a flat bottom alumina boat and transferred to the center of a tube furnace capable of sustaining 1600 °C for long periods of time. The alumina tube was closed at either extreme with a water-cooled steel gauge that permitted an inert atmosphere, N_2_ in this case, to be maintained inside the tube for the full duration of the experiments. These were carried out at 1300 °C over 30 days, 90 days and 180 days, and at 1500 °C over 30 days, which is roughly equivalent to one year at 1300 °C (Fig. 1). Once the experiments were finished, the crucibles were slowly cooled at room temperature and zircon grains released from the silica envelope by dissolving it in warm HF, decanting the fluid and washing the zircon grains first with a saturated H_3_BO_3_ solution and then with ultrapure water to eliminate all traces of acid. The so released zircons grains were dried, mounted in epoxy, documented with the SEM, and analyzed with the SHRIMP as described below. In this way, we recovered between 80% to 40% of the zircon load, depending on how the heated grains behaved: some of them were so broken and cracked that they were useless for SHRIMP analyses.

Once mounted and polished, zircon grains were studied by optical and cathodoluminescent imaging, coated with a 10 nm thick gold layer, and analyzed for U-Th-Pb using a SHRIMP IIe/mc ion microprobe at the IBERSIMS laboratory of the CIC- University of Granada, Spain. The SHRIMP U-Th-Pb analytical method generally followed that described in^41^, and detailed at www.ugr.es/ibersims. Uranium concentration was calibrated using the SL13 reference zircon (U: 238 ppm). U/Pb ratios were calibrated using the TEMORA-II reference zircon (417 Ma^56^) which was measured every 4 unknowns. When required, common lead was corrected from the measured ^204^Pb/^206^Pb, using the model of terrestrial Pb evolution of Cumming and Richards^57^. Data reduction was done with the SHRIMPTOOLS software (downloadable from www.ugr.es/~fbea) using the STATA™ programming language. The SHRIMP results are included as downloadable files.

### Numerical simulations

For these we used COMSOL™, a commercial finite element software that links a given geometry with multiple partial differential equations^58^. To simulate the diffusion of U^4+^ and Pb^2+^ we used Cherniak and Watson^25^ Arrhenius equations: D_Pb_ = 0.0776 e^(−545000/RT)^ m^2^s^−1^ and D_*U*_ = 1.63 e^(−726000/RT)^ m^2^s^−1^ and resolved Fick’s second law: ∂ c_i_/∂ t = ∇. (D_*i*_ ∇ c_*i*_); where c_*i*_ and D_*i*_ are the concentration and the diffusion coefficient of the species *i*, respectively, and t is the time. For the “diffusive” models, we assumed the zircon was surrounded by a material with the same diffusivity as a felsic silicate melt as calculated in^59^: D_*Pb*_ = exp^(−9.08−4.32*w−(28512−7900*w)/T)^ where *w* represents the percentage of water. For the “non-diffusive” models we assumed a D_*Pb*_ three orders of magnitude smaller than in zircon.

Calculations were done for two zircon model geometries that simulate the most common geometries of the tonalite SAB51 and orthogneiss SAB50 zircon grains. For SAB51 we used a 170 × 78 × 56 µm prismatic crystal terminated by a short pyramid at one end and concentrically zoned, with 5 µm wide zones. For SAB50 we used a 160 × 75 × 65 µm prismatic crystal terminated by a short pyramid at either end that contains a large ellipsoidal core with dimensions of 100 × 70 × 56 µm. The core had a concentrically zoned 50 × 35 × 25 µm internal domain also with 5 µm wide zones. In both models the ages were calculated by averaging the concentration of ^238^U, ^206^Pb and ^207^Pb inside a 20 × 17 µm ellipse that simulates the surface analyzed by the SHRIMP ion microprobe with a standard 120 µm Kohler aperture. The ellipse is located in an XY working plane that cuts the crystal in two halves, and randomly moved on that plane within the crystal section always including more than one zone. Details of the procedure can be found in^21^. The COMSOL™ applications, either as COMSOL, Java, Matlab, or VBA model files, are available from F. Bea upon reasonable request.

## Electronic supplementary material

Supplementary information

Dataset 1

Dataset 2

## References

[1] Pilot J, Werner CD, Haubrich F, Baumann N (1998). Palaeozoic and proterozoic zircons from the Mid-Atlantic ridge. Nature.

[2] Andrews GDM (2016). Age and compositional data of zircon from sepiolite drilling mud to identify contamination of ocean drilling samples. Geochemistry, Geophysics, Geosystems.

[3] Efimov, A. A. & Efimova, L. P. The Kytlym platiniferous massif (in Russian) Nedra, Moscow, 336 p (1967).

[4] Neuerburg GJ (1961). A method of mineral separation using hydrofluoric acid. American Mineralogist.

[5] Bea F (2001). Recycling of continental crust into the mantle as revealed by Kytlym Dunite zircons, Urals Mts. Russia. Terranova.

[6] Bortnikov NS (2008). Finds of young and ancient zircons in gabbroids of the Markov Deep, Mid-Atlantic Ridge, 5°54′–5°02.2′N (Results of SHRIMP-II U-Pb Dating): Implication for deep geodynamics of modern oceans. Dokl. Earth Sc..

[7] Skolotnev SG, Bel’Tenev VE, Lepekhina EN (2010). Younger and older zircons from rocks of the oceanic lithosphere in the Central Atlantic and their geotectonic implications. Geotectonics.

[8] Robinson PT (2015). The origin and significance of crustal minerals in ophiolitic chromitites and peridotites. Gondwana Research.

[9] Koglin N, Kostopoulos D, Reischmann T (2009). The Lesvos mafic-ultramafic complex, Greece: Ophiolite or incipient rift. Lithos.

[10] Holis SP (2013). Evolution of the Tyrone ophiolite, Northern Ireland, during the Grampian-Taconic orogeny: a correlative of the Annieopsquotch Ophiolite Belt of central Newfoundland. Journal of the Geological Society.

[11] Xiong F (2016). Diamonds and Other Exotic Minerals Recovered from Peridotites of the Dangqiong Ophiolite, Western Yarlung-Zangbo Suture Zone, Tibet. Acta Geologica Sinica-English Edition.

[12] Krasnobaev AA, Bea F, Fershtater GB, Montero P (2007). The polychronous nature of zircons in gabbroids of the Ural Platinum Belt and the issue of the Precambrian in the Tagil Synclinorium. Doklady Earth Sciences.

[13] Smyth HR, Hamilton PJ, Hall R, Kinny PD (2007). The deep crust beneath island arcs: inherited zircons reveal a Gondwana continental fragment beneath East Java, Indonesia. Earth and Planetary Science Letters.

[14] Stern RJ (2010). Distribution and significance of pre-Neoproterozoic zircons in juvenile Neoproterozoic igneous rocks of the Arabian-Nubian Shield. American Journal of Science.

[15] Rojas-Agramonte Y (2016). Recycling and transport of continental material through the mantle wedge above subduction zones: A Caribbean example. Earth and Planetary Science Letters.

[16] Rojas-Agramonte Y (2017). Ancient xenocrystic zircon in young volcanic rocks of the southern Lesser Antilles island arc. Lithos.

[17] Sánchez-Rodrıiguez L, Gebauer D (2000). Mesozoic formation of pyroxenites and gabbros in the Ronda area (southern Spain), followed by Early Miocene subduction metamorphism and emplacement into the middle crust: U-Pb sensitive high-resolution ion microprobe dating of zircon. Tectonophysics.

[18] Zanetti A (2016). Origin and age of zircon-bearing chromitite layers from the Finero phlogopite peridotite (Ivrea–Verbano Zone, Western Alps) and geodynamic consequences. Lithos.

[19] Watson EB, Cherniak DJ, Hanchar JM, Harrison TM, Wark DA (1997). The incorporation of Pb into zircon. Chemical Geology.

[20] Kramers J, Frei R, Newville M, Kober B, Villa I (2009). On the valency state of radiogenic lead in zircon and its consequences. Chemical Geology.

[21] Bea F, Montero P (2013). Diffusion-induced disturbances of the U-Pb isotope system in pre-magmatic zircon and their influence on SIMS dating. A numerical study. Chemical Geology.

[22] Tanaka K, Takahashi Y, Horie K, Shimizu H, Murakami T (2010). Determination of the oxidation state of radiogenic Pb in natural zircon using X-ray absorption near-edge structure. Physics and Chemistry of Minerals.

[23] Kogawa M, Watson EB, Ewing RC, Utsunomiya S (2012). Lead in zircon at the atomic scale. American Mineralogist.

[24] Cherniak DJ, Watson EB (2001). Pb Diffusion in Zircon. Chemical Geology.

[25] Cherniak DJ, Watson EB (2003). Diffusion in zircon. Reviews in mineralogy and geochemistry.

[26] Cherniak DJ (2010). Diffusion in Accessory Minerals: Zircon, Titanite, Apatite, Monazite and Xenotime. Reviews in Mineralogy & Geochemistry.

[27] Beattie P (1993). The effect of partial melting of spinel peridotite on uranium series disequilibria: constraints from partitioning studies. Earth and Planetary Science Letters.

[28] Dunn T, Sen C (1994). Mineral/matrix partition coefficients for orthopiroxene, plagioclase, and olivine in basaltic to andesitic systems: A combined analytical and experimental study. Geochimica et Cosmochimica Acta.

[29] Adam J, Green TH (2006). Trace element partitioning between mica- and amphibole-bearing garnet lherzolite and hydrous basanitic melt: 1. Experimental results and the investigation of controls on partitioning behavior. Contributions to Mineralogy and Perology.

[30] Kaiser A, Lobert M, Telle R (2008). Thermal stability of zircon (ZrSiO_4_). Journal of the European Ceramic Society.

[31] Pena P, de Aza S (1984). The zircon thermal behaviour: effect of impurities. Part 1. Journal of Material Science.

[32] Anfilogov VN (2015). Stability of zircon in dunite at 1400–1550 °C. Dokl. Earth Sc..

[33] Váczi T (2009). On the breakdown of zircon upon “dry” thermal annealing. Miner Petrol.

[34] Anovitz LM, Blencoe JG (1999). Dry melting of high albite. American Mineralogist.

[35] Zaraisky, G. P. & Balashov, V. N. Thermal decompaction of rocks. *In Fluids in the Crust: Equilibrium and transport properties* (eds Shmulovich, K. I., Yardley, B. W. D. & Gonchar, G. G.) 253–284 (1994).

[36] Audétat A (2015). Characterisation of a Natural Quartz Crystal as a Reference Material for Microanalytical Determination of Ti, Al, Li, Fe, Mn, Ga and Ge. Geostandards and Geoanalytical Research.

[37] Chakraborty D (1978). On the incorporation of metallic impurities in synthetic quartz single crystals. Journal of Crystal Growth.

[38] Cherniak DJ (2010). Diffusion in Quartz, Melilite, Silicate Perovskite, and Mullite. Reviews in Mineralogy & Geochemistry.

[39] Zhang Y, Ni H, Chen Y (2010). Diffusion Data in Silicate Melts. Reviews in Mineralogy & Geochemistry.

[40] Hess JC, Dickinson JE, Rutherford MJ (1980). Solubility of zircon, whitlockite and apatite in lunar basalts and granites. Lunar and Planetary Science.

[41] Dickinson JE, Hess JC (1982). Zircon saturation in lunar basalts and granites. Earth & Planetary Science Letters.

[42] Hauri EH, Wagner TP, Grove TL (1994). Experimental and natural partitioning of Th, U, Pb and other trace elements between garnet, clinopyroxene and basaltic melts. Chemical Geology.

[43] Jones JH, Walker D, Pickett DA, Murrell MT, Beattie P (1995). Experimental investigations of the partitioning of Nb, Mo, Ba, Ce, Pb, Ra, Th, Pa, and U between immiscible carbonate and silicate liquids. Geochim Cosmochim Acta.

[44] Gregoire M, Moine BN, O’Reilly SY, Cottin JY, Giret A (2000). Trace Element Residence and Partitioning in Mantle Xenoliths Metasomatized by Highly Alkaline, Silicate- and Carbonate-rich Melts (Kerguelen Islands, Indian Ocean). Journal of Petrology.

[45] Cherniak DJ, Lanford WA, Ryerson FJ (1991). Lead diffusion in apatite and zircon using ion implantation and Rutheford Backscattering techniques. Geochimica et Cosmochimica Acta.

[46] Cherniak DJ (1995). Diffusion of lead in plagioclase and K-feldspar: An investigation using Rutherford Backscattering and Resonant Nuclear Reaction Analysis. Contrib Mineral Petrol.

[47] Cherniak DJ, Dimanov A (2010). Diffusion in Pyroxene, Mica and Amphibole. Reviews in Mineralogy & Geochemistry.

[48] Ewing RC, Meldrum A, Wang LM, Weber WJ, Corrales LR (2003). Radiation effects in zircon. Reviews in Mineralogy and Geochemistry.

[49] Valley JW (2014). Hadean age for a post-magma-ocean zircon confirmed by atom-probe tomography. Nature Geoscience.

[50] Kusiak MA (2015). Metallic lead nanospheres discovered in ancient zircons. Proceedings of the National Academy of Sciences of the United States of America.

[51] Ono S, Funakoshi, Nakajima Y, Tange Y, Katsura T (2004). Phase transition of zircon at high P-T conditions. Contributions to Mineralogy and Petrology.

[52] Montero P, Bea F, González-Lodeiro F, Talavera C, Whitehouse M (2007). Zircon crystallization age and protolith history of the metavolcanic rocks and metagranites of the Ollo de Sapo Domain in central Spain. Implications for the Neoproterozoic to Early-Paleozoic evolution of Iberia. Geological Magazine.

[53] Montero P, Talevera C, Bea F (2017). Geochemical, isotopic, and zircon (U-Pb, O, Hf isotopes) evidence for the magmatic sources of the volcano-plutonic Ollo de Sapo Formation, Central Iberia. Geologica Acta.

[54] Bea F, Montero P, González-Lodeiro F, Talavera C (2007). Zircon Inheritance Reveals Exceptionally Fast Crustal Magma Generation Processes in Central Iberia during the Cambro-Ordovician. Journal of Petrology.

[55] Montero P, Talavera C, Bea F, González-Lodeiro F, Whitehouse MJ (2009). Zircon geochronology and the age of the Cambro-Ordovician rifting in Iberia. Journal of Geology.

[56] Black LP (2004). Improved ^206^Pb/^238^U microprobe geochronology by the monitoring of a trace-element-related matrix effect; SHRIMP, ID-TIMS, ELA-ICP-MS and oxygen isotope documentation for a series of zircon standards. Chemical Geology.

[57] Cumming GL, Richards JR (1975). Ore lead isotope ratios in a continuously changing Earth. Earth and Planetary Science Letters.

[58] COMSOL Multiphysics. Reference Manual v. 5.2. COMSOL AB, Stockholm, Sweden, www.COMSOL.com (2017).

[59] Perez WA, Dunn AM (1996). Diffusivity of strontium, neodymium, and lead in natural rhyolite melt at 1.0 GPa. Geochimica and Cosmochimica Acta.

