# Supplementary material for: Experimental evidence for the preservation of U-Pb isotope ratios in mantle-recycled crustal zircon grains
Source: Sci Rep. 2018 Aug 27;8:12904. doi: 10.1038/s41598-018-30934-4 (PMC6110838; doi:10.1038/s41598-018-30934-4)
Supplement: Supplementary file 1 — Supplementary information [file 41598_2018_30934_MOESM1_ESM.doc]

**Experimental evidence for the preservation of U-Pb isotope ratios in mantle-recycled crustal zircon grains**

(Supplementary information)

authors: ***Fernando Bea*[[1]](#footnote-2), Pilar Montero^1^, Jose Francisco Molina Palma^1^***

**^1^ Department of Mineralogy and Petrology. Campus Fuentenueva. University of Granada, 18002 Granada, Spain.**

**Samples**

For this work we selected two samples containing abundant unaltered zircon with no, or very little, common lead. The first sample, SAB51, is a Variscan tonalite from the Sanabria zone, in NW Iberia, which contains abundant zircons. Most of them are long narrow prisms (150-300 µm) terminated by short pyramids, transparent, colorless to pinkish, and contain abundant inclusions, mostly albite. Previous SHRIMP work (authors’ unpublished data) revealed that SAB51 zircons are isotopically homogeneous, with a U-Pb age of 318 ± 2 Ma, but chemical heterogeneous, with U ranging from about 230 ppm to 1700 ppm.

The second sample, SAB50, is a Cambro-Ordovician orthogneiss from the Ollo de Sapo formation of the Central Iberian Zone [1]. Rocks from this formation have an abnormally high zircon inheritance so that about 90% of their zircons consist of ca. 485 Ma rims around ca. 605 Ma, in some cases older, cores [1 to 5]. The sample studied here is medium-grained, with a marked augen structure defined by large K-feldspar crystals, and is slightly metamorphosed under lower greenschists facies. It contains abundant zircons. Most of them are short stubby prisms terminated at both ends by long pyramids with total length between 100 and 250 µm. The crystals are transparent, pinkish, with small inclusions and optically noticeable ellipsoidal cores occupying most of the central prismatic body.

In the two cases, zircons were separated from about 15 kg of crushed fresh rock sieved between 300 µm and 50 µm. Separation was done by panning, first in water and then in ethanol. The concentrates were purified first with a Nd magnet, and then by hand picking to remove all traces of other minerals.

Batches of 60-80 grains of each rock were repeatedly analyzed with the SHRIMP before starting the diffusion experiments to ensure they always yielded the same age distribution, i.e., the initial heterogeneity of the zircon loads do not mask the effect of heating. Different batches of the tonalite SAB51 zircons always yielded the same age. Different batches of the orthogneiss SAB50 always yielded the same age in the rims but some minor differences in the cores caused by variable, but always small, fraction of older-than-Ediacaran cores. This variation is not relevant for the experiments because only those grains with cores younger than 800 Ma were considered.

**Diffusion experiments**

Volume diffusion in zircon is practically insensitive to pressure, for this reason the experiments were carried out in open crucibles within a N_2_ atmosphere using the following procedure: cylindric alumina crucibles with a diameter of 12 mm and height of 14 mm were filled with a 5 mm thick bed of fumed silica, on top of which about 100 zircons grains were transferred using a needle. The zircons were covered with another 5 mm thick silica layer, and the whole load was then pressed with a steel piston. The crucibles were placed in a flat bottom alumina boat and transferred to the center of a tube furnace capable of sustaining 1600 ºC for long periods of time. The alumina tube was closed at either extreme with a water-cooled steel gauge that permitted an inert atmosphere to be maintained, N_2_ in this case, inside the tube over the full duration of the experiments. These were carried out at 1300 ºC over 30 days, 90 days and 180 days, and at 1500 ºC over 30 days, which is roughly equivalent to one year at 1300ºC (Fig. 1 in main text). Once finished the experiments, the crucibles were slowly cooled at room temperature and zircons released from the silica envelope by dissolving it in cold hydrofluoric acid. In this way, we recovered between 80% to 40% of the zircon load, depending on how the heated grains behaved. After rinsing to remove all traces of HF, the recovered zircons were dried and mounted on sticky tape for SEM studies of the morphology, and then embedded in epoxy resin and polished to make a "megamount" suitable for SHRIMP U-Th-P and Oxygen isotope analysis [6].

**SHRIMP analysis**

Once mounted and polished, zircon grains were studied by optical and cathodoluminescent imaging, coated with a 10 nm thick gold layer, and analyzed for U-Th-Pb using a SHRIMP IIe/mc ion microprobe at the IBERSIMS laboratory of the CIC- University of Granada, Spain. The SHRIMP U-Th-Pb analytical method roughly followed that described by [7], and is described in detail at www.ugr.es/ibersims. Uranium concentration was calibrated using the SL13 reference zircon (U: 238 ppm). U/Pb ratios were calibrated using the TEMORA-II reference zircon (417 Ma; [8]) which was measured every 4 unknowns. When required, common lead was corrected from the measured ^204^Pb/^206^Pb, using the model of terrestrial Pb evolution of [9]. Data reduction was done with the SHRIMPTOOLS software (downloadable from www.ugr.es/~fbea) using the STATA™ programming language. ***The SHRIMP results are included as downloadable files (Tables 1 and 2)***.

**Numerical simulations**

For these we used COMSOL™, a commercial finite element software that links a given geometry with multiple partial differential equations. To simulate the diffusion of U^4+^ and Pb^2+^ we used [10] Arrhenius equations: D_Pb_ = 0.0776 e^(-545000/RT)^ m^2^s^-1^ and D_U_ = 1.63 e^(-726000/RT)^ m^2^s^-1^ and resolved Fick’s second law: ∂ ci /∂ t = Ñ . (Di Ñ ci); where ci and Di are the concentration and the diffusion coefficient of the species i, respectively, and t is the time.

Calculations were done for two zircon model geometries that simulate the most common geometries of the tonalite SAB51 and orthogneiss SAB50 zircons. For SAB51 we used a 170 x 78 x 56 µm prismatic crystal terminated by a short pyramid at one end and concentrically zoned. For SAB50 we used a 160 x 75 x 65 µm prismatic crystal terminated by a short pyramid at either end that contains a large ellipsoidal core with dimensions of 100 x 70 x 56 µm. The core has a concentrically zoned 50 x 35 x 25 µm internal domain. In both models the ages were calculated by averaging the concentration of ^238^U, ^206^Pb and ^207^Pb inside a 20x17 µm ellipse that simulates the surface analyzed by the SHRIMP ion microprobe with a standard 120 µm Kohler aperture. The ellipse is located in an XY working plane that cuts the crystal in two halves, and randomly moved on that plane within the crystal section. Details of the procedure can be found in [11] . ***The COMSOL™ applications, either as COMSOL, Java, Matlab, or VBA model files, are available from F. Bea upon request.***

# References mentioned in the supplementary material

1. Montero, P., Bea, F., González-Lodeiro, F., Talavera, C. & Whitehouse, M. Zircon crystallization age and protolith history of the metavolcanic rocks and metagranites of the Ollo de Sapo Domain in central Spain. Implications for the Neoproterozoic to Early-Paleozoic evolution of Iberia. *Geological Magazine* **144**, 963-976 (2007).

2. Bea, F., Montero, P., Talavera, C. & Zinger, T. A revised Ordovician age for the oldest magmatism of Central Iberia: U-Pb ion microprobe and LA-ICPMS dating of the Miranda do Douro orthogneiss. *Geologica Acta* **4**, 395-401 (2006).

3. Bea, F., Montero, P., Gonzalez Lodeiro, F. & Talavera, C. Zircon inheritance reveals exceptionally fast crustal magma generation processes in Central Iberia during the Cambro-Ordovician. *Journal of Petrology* **48**, 2327-2339 (2007).

4. Montero, P., Talavera, C., Bea, F., González-Lodeiro, F. & Whitehouse, M. J. Zircon geochronology and the age of the Cambro-Ordovician rifting in Iberia. *Journal of Geology* **117**, 174-191 (2009).

5. Montero, P., Talevera, C. & Bea, F. Geochemical, isotopic, and zircon (U-Pb, O, Hf isotopes) evidence for the magmatic sources of the volcano-plutonic Ollo de Sapo Formation, Central Iberia. *Geologica Acta* **15**, 245-260 (2017).

6. Ickert, R., Hiess, J., Williams, I., Holden, P., Ireland, T., Lanc, P., Schram, N., Foster, J. & Clement, S. Determining high precision, in situ, oxygen isotope ratios with a SHRIMP II: Analyses of MPI-DING silicate-glass reference materials and zircon from contrasting granites. *Chemical Geology* **257**, 114-128 (2008).

7. Williams, I. S. & Claesson, S. Isotopic evidence for the Precambrian provenance and Caledonian metamorphism of high grade paragneisses from the Seve Nappes, Scandinavian Caledonides. II: Ion microprobe zircon U-Th-Pb. *Contribution to Mineralogy and Petrology* **97**, 205-217 (1987).

8. Black, L. P., Kamo, S. L., Allen, C. M., Davis, D. W., Aleinikoff, J. N., Valley, J. W., Mundil, R., Campbell, I. H., Korsch, R. J., Williams, I. S. & Foudoulis, C. Improved 206Pb/238U microprobe geochronology by the monitoring of a trace-element-related matrix effect; SHRIMP, ID-TIMS, ELA-ICP-MS and oxygen isotope documentation for a series of zircon standards. *Chemical Geology* **205**, 115-140 (2004).

9. Cumming, G. L. & Richards, J. R. Ore lead isotope ratios in a continuously changing Earth. *Earth and Planetary Science Letters* **28**, 155-171 (1975).

10. Cherniak, D. J. Diffusion in Accessory Minerals: Zircon, Titanite, Apatite, Monazite and Xenotime. *Reviews in Mineralogy & Geochemistry* **72**, 827-869 (2010).

11. Bea, F. & Montero, P. Diffusion-induced disturbances of the U-Pb isotope system in pre-magmatic zircon and their influence on SIMS dating. A numerical study. *Chemical Geology* **340-350**, 1-17 (2013).

1. *Correspondence to: [fbea@ugr.es](mailto:fbea@ugr.es) [↑](#footnote-ref-2)
